# ^11^C-methionine PET aids localization of microprolactinomas in patients with intolerance or resistance to dopamine agonist therapy

**DOI:** 10.1007/s11102-022-01229-9

**Published:** 2022-05-24

**Authors:** W. A. Bashari, M. van der Meulen, J. MacFarlane, D. Gillett, R. Senanayake, L. Serban, A. S. Powlson, A. M. Brooke, D. J. Scoffings, J. Jones, D. G. O’Donovan, J. Tysome, T. Santarius, N. Donnelly, I. Boros, F. Aigbirhio, S. Jefferies, H. K. Cheow, I. A. Mendichovszky, A. G. Kolias, R. Mannion, O. Koulouri, M. Gurnell

**Affiliations:** 1 Cambridge Endocrine Molecular Imaging Group, Metabolic Research Laboratories, Wellcome–MRC Institute of Metabolic Science, University of Cambridge and National Institute for Health Research Cambridge Biomedical Research Centre, Addenbrooke’s Hospital, Cambridge Biomedical Campus, Cambridge, UK; 2 Department of Nuclear Medicine, University of Cambridge and National Institute for Health Research Cambridge Biomedical Research Centre, Addenbrooke’s Hospital, Cambridge Biomedical Campus, Cambridge, UK; 3 Macleod Diabetes and Endocrine Centre, Royal Devon and Exeter Hospital, Exeter, UK; 4 Department of Radiology, Addenbrooke’s Hospital, Cambridge Biomedical Campus, Cambridge, UK; 5 Department of Neuropathology, Addenbrooke’s Hospital, Cambridge Biomedical Campus, Cambridge, UK; 6 Department of Otolaryngology, Addenbrooke’s Hospital, Cambridge Biomedical Campus, Cambridge, UK; 7 Department of Neurosurgery, Addenbrooke’s Hospital, Cambridge Biomedical Campus, Cambridge, UK; 8 Wolfson Brain Imaging Centre, University of Cambridge, Addenbrooke’s Hospital, Cambridge Biomedical Campus, Cambridge, UK; 9 Department of Oncology, Addenbrooke’s Hospital, Cambridge Biomedical Campus, Cambridge, UK

**Keywords:** ^11^C-methionine PET, Microprolactinoma, Dopamine agonist intolerance/ resistance

## Abstract

**Purpose:**

To assess the potential for ^11^C-methionine PET (Met-PET) coregistered with volumetric magnetic resonance imaging (Met-PET/MR^CR^) to inform clinical decision making in patients with poorly visualized or occult microprolactinomas and dopamine agonist intolerance or resistance.

**Patients and methods:**

Thirteen patients with pituitary microprolactinomas, and who were intolerant (*n *= 11) or resistant (*n *= 2) to dopamine agonist therapy, were referred to our specialist pituitary centre for Met-PET/MR^CR^ between 2016 and 2020. All patients had persistent hyperprolactinemia and were being considered for surgical intervention, but standard clinical MRI had shown either no visible adenoma or equivocal appearances.

**Results:**

In all 13 patients Met-PET/MR^CR^ demonstrated a single focus of avid tracer uptake. This was localized either to the right or left side of the sella in 12 subjects. In one patient, who had previously undergone surgery for a left-sided adenoma, recurrent tumor was unexpectedly identified in the left cavernous sinus. Five patients underwent endoscopic transsphenoidal selective adenomectomy, with subsequent complete remission of hyperprolactinaemia and normalization of other pituitary function; three patients are awaiting surgery. In the patient with inoperable cavernous sinus disease PET-guided stereotactic radiosurgery (SRS) was performed with subsequent near-normalization of serum prolactin. Two patients elected for a further trial of medical therapy, while two declined surgery or radiotherapy and chose to remain off medical treatment.

**Conclusions:**

In patients with dopamine agonist intolerance or resistance, and indeterminate pituitary MRI, molecular (functional) imaging with Met-PET/MR^CR^ can allow precise localization of a microprolactinoma to facilitate selective surgical adenomectomy or SRS.

**Supplementary Information:**

The online version contains supplementary material available at 10.1007/s11102-022-01229-9.

## Introduction

Prolactinomas are the most common functioning pituitary adenomas [1]. Microprolactinomas typically manifest with galactorrhea and hypogonadism [2, 3], which can have significant adverse effects on quality of life [4]. The mainstay of treatment remains medical therapy with dopamine agonists [5]. These are generally well tolerated [6], but may cause side effects including postural dizziness, daytime somnolence, gastro-intestinal upset and cardiac valvular fibrosis [7–9], although risk of the latter when using low dosages (as is typically required for prolactinomas) is still debated [10]. In recent years, attention has also focussed on potential psychological adverse effects, including impulse control disorders (ICDs), which have been reported in 8–24% of patients with prolactinomas receiving treatment with dopamine agonists [11–15], and which can have devastating consequences for patients and their families [16].

When adverse effects prevent a successful treatment trial (either with respect to drug dosage and/or duration of therapy), patients are considered *intolerant* to dopamine agonist therapy [3]. This should be distinguished from dopamine agonist *resistance*, which is preferred when there is failure to normalize serum prolactin and/or achieve significant tumor shrinkage (in macroprolactinomas) despite good tolerance and concordance with standard clinical dosages [17, 18]. For patients with intolerance or resistance to medical therapy, transsphenoidal surgery (TSS) is an alternative treatment option [5], with several recent reports suggesting a higher long-term remission rate [19–24] and improved cost-effectiveness compared with dopamine agonist therapy [25, 26]. As evidence for the efficacy and safety of transsphenoidal surgery (TSS) accrues, there is increasing discussion about the earlier deployment of surgery for selected cases [27, 28]. However, even in experienced hands, TSS may be complicated by cerebrospinal fluid leakage and new-onset hypopituitarism (including diabetes insipidus) [19]. Careful preoperative appraisal must therefore balance the probability of achieving surgical cure with these risks. High quality magnetic resonance imaging (MRI) of the sella and parasellar regions is central to effective decision-making and may provide important information regarding the likelihood of achieving complete surgical resection [29]. Nonetheless, it is not always possible to reliably localize the causative microadenoma, and MRI findings may be considered equivocal or negative even prior to a trial of medical therapy [30, 31].

Molecular (functional) imaging using positron emission tomography-computed tomography (PET-CT) can aid localization of de novo, residual or recurrent pituitary adenomas and has been successfully used to facilitate curative (including repeat) TSS in acromegaly and Cushing Disease when MRI is indeterminate [32–34]. Several PET radiotracers have been trialled for imaging prolactinomas, including ^11^C-raclopride and ^11^C-N-methylspiperone (dopamine D2 receptor ligands) [35, 36], ^18^F-fluorodeoxyglucose (metabolic activity as per the *Warburg effect*) [37, 38], and ^11^C-methionine [taken up via the L-type amino acid transporter 1 (LAT1) at sites of peptide synthesis] [38–41]. ^11^C-methionine PET (Met-PET) has been reported to have high sensitivity for the detection of functioning pituitary adenomas, including prolactinomas [38, 42]. Moreover, compared to other pituitary adenoma subtypes, prolactinomas show particularly avid ^11^C-methionine uptake [41, 43]. We have therefore reviewed our recent experience with Met-PET coregistered with volumetric MRI (Met-PET/MR^CR^) in patients with suspected microprolactinomas who are being considered for pituitary surgery due to intolerance or resistance to dopamine agonist therapy, but in whom standard clinical MRI has not conclusively identified a discrete lesion. Here, we show that functional imaging can confirm or refute the suspected site of a microprolactinoma queried on clinical MRI and reveal the location of an adenoma when MRI is negative.

## Patients and methods

### Patients

Thirteen patients with microprolactinomas were referred to our tertiary center for consideration of surgery, because of dopamine agonist intolerance (*n *= 11) or resistance (*n *= 2), between April 2016 and March 2020. In all cases, the diagnosis of a prolactinoma was originally based on typical symptoms (e.g. galactorrhea and/or gonadal dysfunction) in the presence of confirmed raised serum prolactin levels (females > 29 ng/ml, males > 18 ng/ml). Conventional pituitary MRI (T1 spin echo with and without intravenous contrast and, where available, T2 fast spin echo) was deemed equivocal (one or more possible abnormalities identified, but low confidence to confirm site of the adenoma) or negative (no abnormality seen). Each patient underwent Met-PET and volumetric MRI with co-registration to yield hybrid images (Met-PET/MR^CR^) as described in the following sections. The study received institutional approval (CUH QSIS 2020: 3039).

### Clinical care

Patients were managed according to local approved pituitary care pathways, which are consistent with international clinical guidelines [5]. Pituitary function tests [including prolactin, cortisol, free thyroxine (FT4), thyroid stimulating hormone (TSH), luteinizing hormone (LH), follicle stimulating hormone (FSH), estrogen or testosterone, and insulin-like growth factor 1 (IGF-1)] were performed on serum samples collected between 8 and 9AM. All biochemical measurements (Siemens Medical Solutions Diagnostics Ltd.) were performed in a Clinical Pathology Accreditation Ltd. laboratory (CPA) with relevant internal and external quality assurance as required by the CPA. Each patient provided informed consent for Met-PET. All patients remained off dopamine agonist therapy for at least one month prior to performing functional imaging to minimise the risk of a false negative scan due to residual suppression of tumor activity. Treatment decisions were made on a case-by-case basis, considering patient preference, after discussion by a specialist pituitary multidisciplinary team consisting of pituitary neurosurgeons, endocrinologists, otolaryngologists, radiation oncologist, neuropathologist, and neuroradiologists, who had full access to the Met-PET/MR^CR^ scans to inform clinical decision-making. Transsphenoidal pituitary surgery or radiotherapy were performed as previously described [34, 44].

### Synthesis of ^11^C-methionine

The PET tracer, L-[methyl-^11^C]-methionine, was synthesised in compliance with good manufacturing practice using a captive solvent in loop methylation method without preparative HPLC, adapted from methods published previously [45–47]. Briefly, [^11^C]CO_2_ was produced using a PETtrace cyclotron (GE Healthcare, Milwaukee, WI, USA) via the ^14^ N(p,$\alpha$)^11^C reaction before conversion to [^11^C]MeI in the MeI MicroLab (GE Healthcare). This was then transferred to the HPLC loop of a modified TracerLabFXC (GE Healthcare) synthesiser containing an L-homocysteine precursor solution (0.5 M aqueous NaOH solution in ethanol). ^11^C-methionine was produced in yields averaging 376 MBq with a radiochemical purity of > 96% and specific activity between 263 and 452.5 MBq.

### ^11^C-methionine PET-CT imaging

Images were acquired on a GE Discovery 690 PET-CT scanner (GE Healthcare). All patients fasted for 4 h before PET-CT scanning. An intravenous injection of 264–423 MBq of L-[methyl-^11^C]-methionine was given prior to each scan. The uptake time for PET-CT was standardized at 20 min. An attenuation correction (low dose) CT was performed (140 kV, 220 mA, 0.5 s rotation, and 0.984 mm pitch) followed by a single bed position PET acquisition of the head. Time-of-flight (ToF) PET data were acquired for a total acquisition time of twenty minutes. PET images were reconstructed with CT attenuation correction using fully 3D iterative reconstruction algorithms (three iterations, 24 subsets, 2 mm Gaussian post-filter) incorporating ToF and resolution recovery software (VUE Point FX and Sharp IR) to a 3.27 mm slice thickness. CT images were reconstructed at 1.25 mm slice thickness. Met-PET studies were independently reviewed by nuclear medicine physicians with expertise in PET-CT on the Xeleris workstation (GE Healthcare, Amersham, Buckinghamshire, UK).

### Standard and 3D gradient echo MRI

MR imaging was performed on clinical 1.5 T or 3 T systems (GE Healthcare, Waukesha, WI, USA) using a circularly polarised head coil. Coronal T1 spin echo (SE) images were obtained before and after intravenous injection of 0.1 mmol/kg gadobutrol. A fast spoiled gradient recalled echo (FSPGR) acquisition sequence was performed to optimise co-registration with the PET-CT dataset (Met-PET/MR^CR^). In brief, sagittal T1-weighted FSPGR images [TR (repetition time) 11.5 ms, TE (echo time) 4.2 ms, slice thickness 1 mm, 0 mm gap, 256 × 256 matrix] of the whole head were obtained following intravenous injection of 0.1 mmol/kg gadobutrol.

### Image processing and analysis

Image processing was performed using open source software 3D Slicer [48] (version 4.10.2, 05–2019). PET images (GE SharpIR reconstruction) were prepared for visualization by creating ratio PET (SUVr) images. SUVr images were created by dividing each voxel in the image by the mean value found in a region of interest (ROI) positioned in the subject’s cerebellum. Each subject’s SUVr images were displayed with identical colour scales (ColdToHotRainbow), colour ranges (1.0–4.0), and threshold levels to remove low level background uptake (< 1.0). SUVr images were registered with volumetric MRI images (FSPGR sequence) using rigid registration with 6 degrees of freedom, a maximum number of iterations of 1500 and a sample ratio of 0.01. Following registration, the SUVr images were overlaid on the MRI images.

## Results

**Fig. 1:**
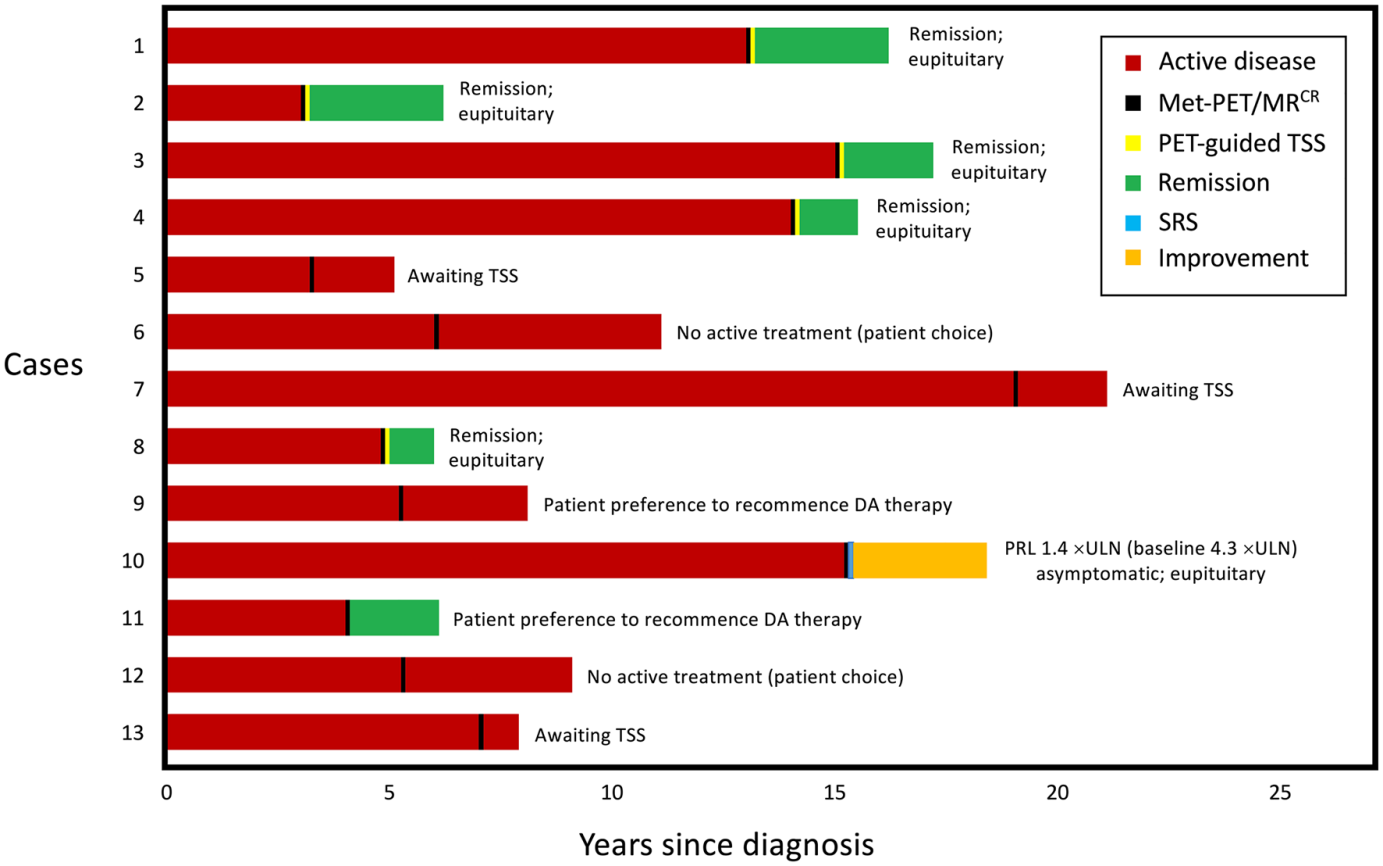
Schematic representation of the clinical courses for each of the thirteen patients prior to and following Met-PET. *DA* dopamine agonist,* Met-PET/MR*^*CR*^ ^11^C-methionine PET coregistered with volumetric (FSPGR) MRI, *PET* Positron Emission Tomography, *PRL* prolactin, *SRS* stereotactic radiosurgery, *TSS* transsphenoidal surgery, *ULN* upper limit of normal

**Fig. 2:**
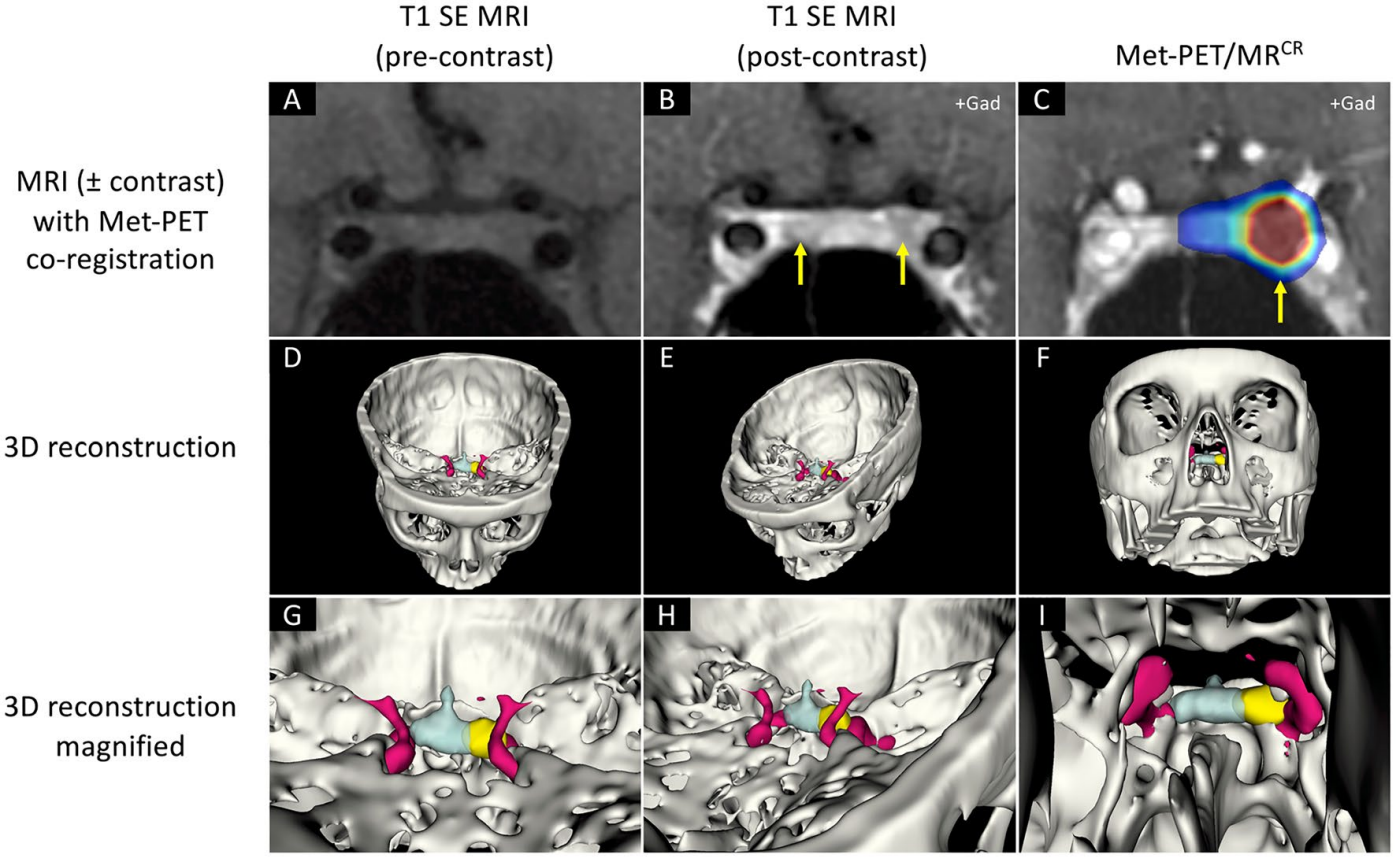
MRI and Met-PET findings with 3D reconstruction of the sella and parasellar regions in case 1. **A–B** Pre- and post-contrast coronal T1 SE MRI demonstrates equivocal appearances, with two possible areas of reduced enhancement (arrows). **C** Met-PET/MR^CR^ reveals avid focal tracer uptake in the left side of the gland adjacent to the cavernous sinus (arrow). **D–I** 3D reconstructed images, combining PET, CT and FSPGR MRI datasets, allows appreciation of the location of the tumor (yellow) with respect to the normal gland (turquoise) and proximity of the tumor to key adjacent structures including the intracavernous cartoid artery (red). At transsphenoidal surgery, a microadenoma abutting the left cavernous sinus was resected and confirmed histologically to be a prolactinoma. Postoperatively the patient remains normoprolactinemic and eupituitary. *CT* computed tomography, *FSPGR* fast spoiled gradient recalled echo, *Gad* gadolinium, *MRI* magnetic resonance imaging,* Met-PET/MR*^*CR*^ ^11^C-methionine PET-CT coregistered with volumetric (FSPGR) MRI, *PET* positron emission tomography, *SE* spin echo

**Fig. 3:**
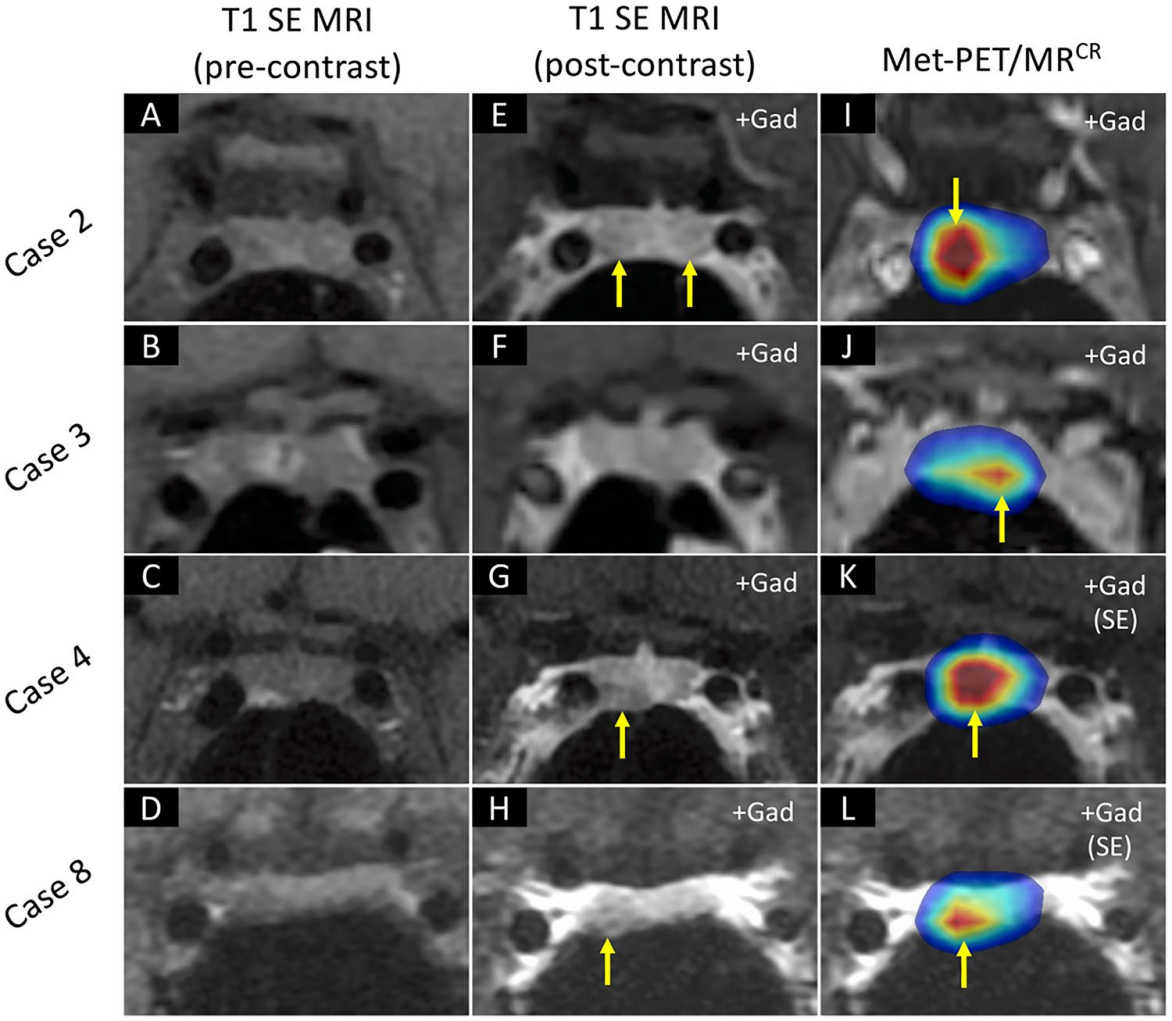
MRI and Met-PET findings in cases 2, 3, 4 and 8. **A–H** Pre- and post-contrast coronal T1 SE MRI show equivocal appearances in four patients, identifying either no abnormality or possible single or multiple lesions (arrows). **I–L** In contrast, in all four subjects Met-PET/MR^CR^ demonstrates a single focus of intense tracer uptake which was subsequently confirmed at transsphenoidal surgery to be the site of a microprolactinoma. Postoperatively, all patients remain normoprolactinemic and eupituitary. *FSPGR* fast spoiled gradient recalled echo, *Gad* gadolinium, *MRI* magnetic resonance imaging,* Met-PET/MR*^*CR*^ ^11^C-methionine PET-CT coregistered with volumetric (FSPGR) or SE MRI, *PET* positron emission tomography, *SE* spin echo

**Fig. 4:**
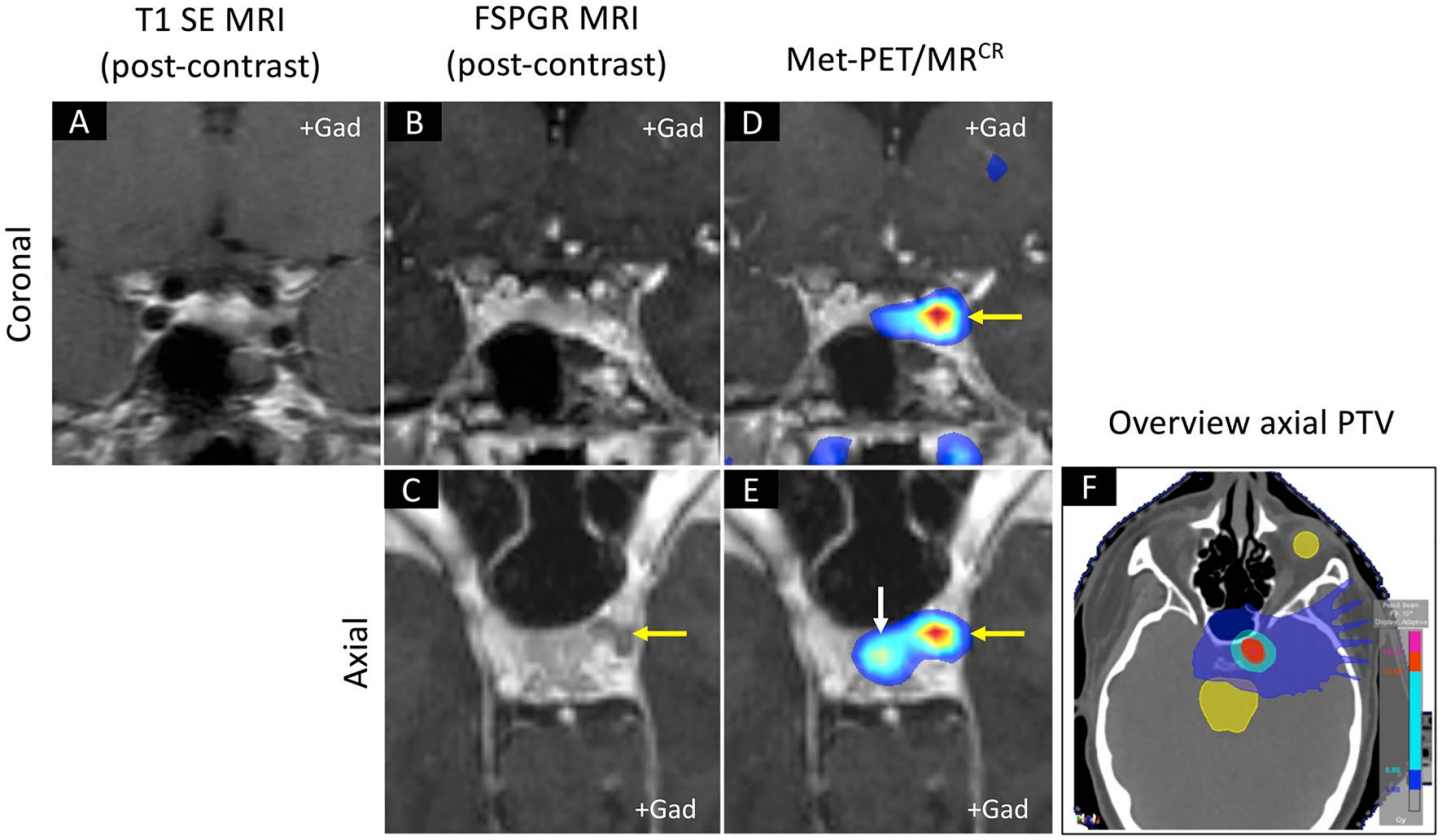
PET–guided stereotactic radiosurgery in case 10. **A–B** Post-contrast coronal T1 SE and FSPGR MRI demonstrate indeterminate appearances in a patient who had previously undergone transsphenoidal surgery for a left-sided microprolactinoma. **C** Axial FSPGR MRI shows possible recurrent tumor in the left cavernous sinus (yellow arrow). **D–E** Coronal and axial Met-PET/MR^CR^ confirm avid tracer uptake at the site of the suspected recurrence (yellow arrow); tracer uptake within the remaining normal gland is also seen (white arrow). **F** Treatment plan for PET-guided SRS. Three years later serum prolactin was near-normalized (1.4 × ULN). *FSPGR* fast spoiled gradient recalled echo, *Gad* gadolinium, *MRI* magnetic resonance imaging, *Met-PET/MR*^*CR*^ ^11^C-methionine PET-CT coregistered with volumetric (FSPGR) MRI, *PET* positron emission tomography, *PTV* Planning Target Volume, *SE* spin echo, *ULN* upper limit of normal

Thirteen patients, all of reproductive age, were included in the study [twelve women, one man; mean age at time of Met-PET scan 34 years (range 20–45)]. Eleven patients were referred for Met-PET because of intolerance to DA therapy, and two because of DA resistance (Table 1). All had experienced several years (in some instances > 10 years) of suboptimal disease control (Fig. 1). In seven subjects (Cases 2, 5, 7, 8, 11, 12, 13), findings on pituitary MRI at the time of referral to our service were similar to those reported at initial presentation (Table 1). In three patients initial MRI appearances were either less informative regarding the suspected site of the adenoma (Cases 3, 9) or incorrectly identified a possible abnormality on the contralateral side to where the adenoma was subsequently resected (Case 1) (Table 1). In a single patient (Case 6), MRI at diagnosis identified a possible adenoma that was not readily visualized on repeat MRI at the time of referral for Met-PET (Table 1). In two patients (Cases 4, 10), initial imaging was unavailable for review. Met-PET identified a focus of increased tracer uptake in all thirteen cases (Figs. 2–4 and Supplementary Fig. 1). Five patients underwent uncomplicated PET-guided TSS with intra-operative and, in four cases, histological confirmation of PET findings. All had subsequent complete remission of hyperprolactinemia, which has been maintained off medical treatment, and all have normal pituitary function (Figs. 1–3). Three patients are awaiting surgery. One patient was deemed to have unresectable lateral disease, and therefore received stereotactic radiosurgery (SRS), with subsequent near normalization of hyperprolactinemia (Table 1; Figs. 1 and 4). Four patients had a clear abnormal focus of tracer uptake on Met-PET but chose not to undergo surgical intervention after further consideration of the risks and benefits of surgery (Table 1; Supplementary Fig. 1). Two of these patients have returned to cabergoline therapy despite ongoing side effects, with one achieving a normal serum prolactin level, while two patients have elected to remain off all treatment with ongoing hyperprolactinemia (Fig. 1).

**Table 1:** Clinical, biochemical and radiological features at initial presentation, at the time of Met-PET, and following further treatment

Case	Sex	Baseline biochemistry		MRI findings at diagnosis	Previous treatment	DA side effects or resistance	MRI findings following previous treatment	Met-PET/MR^CR^ findings	PRL at time of PET (ng/ml) ^*^	Further treatment	Biochemistry following further treatment		Latest PRL (ng/ml)^*^
		PRL (ng/ml)^*^	Pituitary deficits								PRL (ng/ml)^*^	Pituitary deficits	
1	F	203	G	Infundibulum central; possible right-sided lesion	C, B, Q	Low mood	Infundibulum central; possible bilateral non-enhancing lesions	Focal high tracer uptake adjacent to left CS	107	TSS	10	None	24
2	F	67	G	Subtle left infundibular deviation; possible bilateral non-enhancing lesions	C	Low mood	Subtle left infundibular deviation; possible bilateral non-enhancing lesions	Focal high tracer uptake within right sella	74	TSS	5	None	5
3	F	172	G	Possible right infundibular deviation; no discrete lesion	C, B, Q	Low mood, headache	Right infundibular deviation; no discrete lesion	Focal high tracer uptake just to left of infundibulum inferiorly	73	TSS	15	None	8
4	F	48	G	Unavailable	C, Q	Low mood	Infundibulum central; possible right sided lesion with subtle depression of sella floor	Focal high tracer uptake within right sella	49	TSS	3	None	6
5	F	109	None	Infundibulum central; no discrete lesion	C, A**	Low mood	Infundibulum central; no discrete lesion	Focal high tracer uptake within right sella	100	Awaiting TSS	NA	NA	100
6	M	191	G	Infundibulum central; possible left-sided lesion	C, B, Q	Aggression, increased libido	Infundibulum central; no discrete lesion	Focal high tracer uptake within left sella	61	Nil	NA	G	46
7	F	52	None	Infundibulum central; minor depression of left sella floor; no discrete lesion	C, B, Q	Low mood	Infundibulum central; minor depression of left sella floor; no discrete lesion	Focal high tracer uptake within sella inferiorly to the left of midline	49	Awaiting TSS	NA	N/A	49
8	F	56	G	Infundibulum central; minor inferior depression of right sella floor; possible right microadenoma	C, Q	Nausea	Infundibulum central; minor inferior depression of right sella floor; possible right microadenoma	Focal high tracer uptake within right sella inferiorly	36	TSS	6	None	6
9	F	75	G	Infundibulum central; no discrete lesion	C	Headache	Infundibulum central; possible area of reduced enhancement in left side of sella	Focal high tracer uptake within left sella	46	C	29	None	35
10	F	470	G	Unavailable; presumed left-sided adenoma (site of previous TSS)	C, TSS	Raynaud phenomenon	Post-operative changes; no definite residual/recurrence	Focal high tracer uptake within left CS	82	SRS	86	None	40
11	F	65	G	Infundibulum central; possible bilateral non-enhancing lesions	C	Nausea, increased libido	Infundibulum central; possible bilateral non-enhancing lesions	Focal high tracer uptake within right sella	55	C	15	None	8
12	F	120	G	Possible subtle left Infundibular deviation; no discrete lesion seen	C	Resistance	Possible subtle left Infundibular deviation; no discrete lesion seen	Focal high tracer uptake within left sella	111	Nil	NA	NA	35
13	F	84	G	Possible subtle left Infundibular deviation with right-sided lesion in superior aspect of gland	C	Resistance	Possible subtle left Infundibular deviation with right-sided lesion in superior aspect of gland	Focal high tracer uptake within right sella	39	Awaiting TSS	NA	NA	37

*Prolactin references ranges: female (3–29 ng/ml), male (2–18 ng/ml)

**Treated with aripiprazole for a concomitant mental health condition

*A* aripiprazole, *B* bromocriptine, *BP* blood pressure, *C* cabergoline, *CS* cavernous sinus, *DA* dopamine agonist, *F* female, *G* hypogonadism, *M* male, *NA* not available, *PRL* prolactin, *Q* quinagolide, *SRS* stereotactic radiosurgery, *TSS* transsphenoidal surgery (endoscopic)

The five patients who underwent selective adenomectomy and the single patient who underwent SRS, guided by the findings on Met-PET, are presented in more detail below.

### Case 1 (Table 1 and Figs. 1–2)

A young woman presented with secondary amenorrhea and was found to have significant hyperprolactinemia (serum prolactin 203 ng/ml). Initial and subsequent MRI did not identify a clear adenoma, although a possible right-sided lesion was queried. Over the course of 15 years, several DAs were trialled, including bromocriptine (maximum tolerated dosage 5 mg/day), cabergoline (0.5 mg/week), and quinagolide (75 μg/day). Normoprolactinaemia was never achieved, and the patient experienced recurrent episodes of low mood while on treatment (Fig. 1). Repeat T1 SE MRI remained equivocal, highlighting possible abnormalities on both sides of the gland (Fig. 2). Met-PET identified a focus of intense ^11^C-methionine uptake in the left lateral sella (Fig. 2). The patient proceeded to endoscopic TSS, with selective resection of a left-sided tumour which was histologically confirmed as a lactotroph pituitary adenoma. She remains in remission off all treatment at 3 years follow-up, with otherwise normal pituitary function.

### Case 2 (Table 1 and Fig. 3)

A young woman developed secondary amenorrhea, bilateral galactorrhea, and low libido. Serum prolactin was raised at 67 ng/ml. T1 gadolinium-enhanced SE and FSPGR MRI failed to demonstrate a convincing adenoma, although possible focal reduced contrast enhancement was queried bilaterally (Fig. 3). Cabergoline therapy (maximum tolerated dosage 0.75 mg/week) was associated with depressive symptoms and failure to normalize serum prolactin (Fig. 1). Met-PET revealed a focus of intense ^11^C-methionine uptake in the right side of the pituitary gland. The patient underwent endoscopic TSS with selective resection of a right-sided lactotroph adenoma (with confirmatory histology). She remains in remission 3 years later, with normal pituitary function.

### Case 3 (Table 1 and Fig. 3)

A young woman was found to have a raised serum prolactin level (172 ng/ml) during investigation for secondary amenorrhea. A diagnosis of a presumed microprolactinoma was made, although T1 gadolinium-enhanced SE MRI did not identify a discrete lesion. Trials of cabergoline (0.5 mg/week), bromocriptine (10 mg/day) and quinagolide (150 µg/day) each allowed restoration of normoprolactinemia, but all resulted in intolerable side effects with low mood and headaches. Repeat T1 gadolinium-enhanced SE MRI showed subtle infundibular deviation to the right but no discrete lesion (Fig. 3). Met-PET revealed a focus of high tracer uptake inferiorly and just to the left of midline (Fig. 3), which corresponded with a small area of abnormal tissue at TSS. Although histology was unable to confirm an adenoma (insufficient sample), immediately following surgery the patient’s serum prolactin was normal, and she remains in remission 2 years after surgery with no pituitary hormone deficits.

### Case 4 (Table 1 and Fig. 3)

A young woman with secondary amenorrhea was found to have mild hyperprolactinemia (serum prolactin 48 ng/ml). The findings from initial T1 gadolinium-enhanced SE MRI were unavailable for review. The patient was commenced on cabergoline but was unable to tolerate even the lowest dosage (0.25 mg/week) due to mood disturbance. Thereafter, quinagolide (75 µg/day) was tried but resulted in return of depressive symptoms and the patient elected to discontinue DA therapy. Several years later, she sought further advice about possible surgical treatment of her prolactinoma given her persistent symptoms and ongoing hyperprolactinemia. Repeat T1 gadolinium-enhanced SE MRI identified a possible right-sided pituitary microadenoma (Fig. 3). Whilst surgery could have been undertaken based on these MRI findings alone, after discussion with the patient molecular imaging was performed to increase confidence that the suspected lesion was indeed functioning. Met-PET showed intense ^11^C-methionine tracer uptake within the right side of the sella (Fig. 3). At TSS, a right-sided adenoma was resected, with histological confirmation of a lactotroph adenoma. Thereafter, serum prolactin levels have normalized, with restoration of regular menses (and maintained for > 12 months).

### Case 8 (Table 1 and Fig. 3)

A young woman developed oligomenorrhea and bilateral galactorrhea. Serum prolactin was raised at 56 ng/ml. Initial T1 gadolinium-enhanced SE MRI was considered suggestive of a possible right-sided pituitary microadenoma, with subtle depression of the sella floor. Cabergoline (0.5 mg/week) was initiated; however, she developed significant nausea, which did not improve despite changing to quinagolide (75 μg/day). Her symptoms recurred on subsequent rechallenging with dopamine agonist therapy and surgery was therefore considered. Repeat T1 SE MRI identified a suspected lesion in the right side of the pituitary gland (Fig. 3). The possibility of proceeding direct to surgery based on the MRI findings alone was considered but, following discussion with the patient, Met-PET was performed to confirm a functioning lesion at this location. This demonstrated focal increased ^11^C-methionine uptake in the anterior-inferior aspect of the pituitary fossa, concordant with the site suspected on MRI (Fig. 3). The patient underwent PET-guided TSS, with histological confirmation of a prolactinoma at this location. She remains in remission postoperatively (at 12 months), with otherwise normal pituitary function.

### Case 10 (Table 1 and Fig. 4)

A young woman was found to have hyperprolactinemia (serum prolactin 470 ng/ml) while being investigated for secondary amenorrhea. The findings of MRI at initial presentation were not available. She was treated with cabergoline in increasing dosages (up to 4 mg/week), with varying control of hyperprolactinemia. During this time, the patient developed marked Raynaud phenomenon and dopamine agonist therapy was discontinued. She then proceeded to TSS, and a left-sided lactotroph microadenoma was resected (confirmed histologically). Following a short period of normoprolactinemia, her symptoms returned, and serum prolactin was again elevated (82 ng/ml). However, T1 gadolinium-enhanced SE MRI could not reliably identify recurrent adenoma tissue (Fig. 4). Unexpectedly, Met-PET revealed a small focus of avid 11C-methionine uptake within the left cavernous sinus (Fig. 4). A small hypointense abnormality could be appreciated at exactly the same location on axial FSPGR MRI (Fig. 4). As the recurrent tumour was considered inoperable, the patient underwent SRS. Prolactin levels have decreased to 40 ng/ml, at 3 years following SRS, with no new pituitary deficits.

## Discussion

We report our initial experience with Met-PET/MR^CR^ in 13 patients with microprolactinomas and dopamine agonist intolerance or resistance, in whom standard clinical MRI was considered indeterminate or negative. In all 13 cases, Met-PET demonstrated a focus of increased (often intense) tracer uptake (Figs. 2–4 and Supplementary Fig. 1). In some instances, this correlated with an area that had been identified on MRI as possibly in keeping with an adenoma, but in other subjects Met-PET did not support MRI findings and/or revealed a previously undisclosed abnormality (Table 1; Figs. 1–4 and Supplementary Fig. 1). In all five patients who proceeded to surgery, complete and sustained biochemical remission was achieved, often for the first time in many years, with histology confirming a lactotroph adenoma in four cases. In the fifth patient, an obvious abnormality was found at surgery at the site identified on Met-PET, but histology was not confirmatory of a prolactinoma; however, this likely reflected a small tumor with total resection as evidenced by restoration and maintenance of normal serum prolactin following surgery—analogous to surgical/histological findings in some corticotroph tumors. In the patient with recurrent hyperprolactinemia following previous TSS (Case 10), in whom recurrent tumor was localized within the left cavernous sinus, SRS was followed by a progressive fall in serum prolactin to near normal levels (1.4 × ULN) (Table 1 and Figs. 1 and 4). Importantly, in all patients undergoing surgery, normal pituitary function was maintained or restored, and there were no other surgical complications.

Traditonally, dopamine agonist therapy has been considered the cornerstone of management of patients with prolactinoma [5, 49]. In particular, cabergoline is recommended as it has superior efficacy in achieving normoprolactinemia and tumour shrinkage, when compared with bromocriptine and quinagolide. However, two important factors merit consideration before embarking on medical therapy: (1) the potential need for long-term treatment and (2) possible adverse effects of dopamine agonist therapy. With respect to treatment duration, two recent systematic reviews concluded that following withdrawal of medical therapy, which is usually undertaken after two years of treatment, only approximately one-third of patients will achieve sustained remission [50, 51]. As a result, many patients require long-term (even > 10 years) treatment [16]. Although dopamine agonists are generally considered safe, a longer duration of treatment means that there is an extended exposure window in which the patient may experience side effects, and which may have particular relevance, for example, when considering the risk of cardiac valvular fibrosis [8, 10]. In addition, in recent years, concerns have surfaced regarding the possible adverse psychological effects, and in particular the previously unrecognized high prevalence of impulse control disorders (ICDs), in those treated with dopamine agonists [11–15, 52], which were not fully appreciated when earlier guidelines were published [5]. Accordingly, recent guidelines acknowledge that surgery can be considered as a first line treatment option for microprolactinomas where complete resection is deemed possible following specialist neurosurgical evaluation [53].

In support of this, several groups have reported on the effectiveness and safety of prolactinoma surgery [21–24, 29, 54–62]: after a follow-up of 13.5–102 months, overall remission rates ranged from 26 to 92%, with most estimates around 70%, although not all studies have provided clarity on whether patients required ongoing dopamine agonist therapy to achieve postoperative remission. Not surprisingly, most studies have reported higher remission rates for microprolactinomas compared to macroprolactinomas, and adenomas that are enclosed within the gland may have a more favourable outcome compared with adenomas located at the lateral margins [29, 60]. These findings have been endorsed in several systematic reviews and meta-analyses, which have reported TSS to deliver superior clinical outcomes compared to dopamine agonist therapy [19, 20, 63], with superior cost-effectiveness, although the absence of any randomised trials remains a major limitation [19]. Interestingly, one systematic review specifically investigated prolactinoma patients undergoing surgery because of resistance or intolerance to dopamine agonists, or patient preference, and reported that 38% achieved sustained remission without further treatment (66% of microprolactinomas, 22% of macroprolactinomas), while 62% achieved remission with adjunctive dopamine agonist therapy [64].

As the majority of prolactinomas are microadenomas [49], selective and complete adenomectomy, which delivers long-term remission without causing additional pituitary deficits (and where possible correcting exisiting deficits), should be the goal of transsphenoidal surgery. This is particularly important for young women considering reproduction, who are the group most commonly affected by microprolactinomas. To facilitate selective adenomectomy, accurate preoperative localisation of the adenoma is important, to minimise the need for more extensive exploration of the gland, and thereby potentially reducing the risk of new pituitary deficiencies or other (e.g. neurovascular) complications. Nonetheless, even with advances in MR imaging, the detection of microadenomas, especially < 3 mm in maximum diameter, remains challenging [65]. Additionally, the finding of an incidentaloma may confound management [66]. In these situations, Met-PET may offer an additional route to confirming/revealing the tumor, as exemplified by the cases reported in our cohort, and also in other pituitary tumor subtypes [32, 33]. In this way, Met-PET complements routine clinical MRI to improve the accurate localization of small functioning tumours, and thereby enable patients who might otherwise not be considered suitable candidates for surgery or radiotherapy to progress to TSS or SRS.

Our findings are also consistent with previous reports that indicate microprolactinomas are particularly ^11^C-methionine-avid [41, 43]. Met-PET may therefore allow more reliable distinction between true microprolactinomas and coincidental small non-secretory adenomas, although formal studies would be required to confirm this. It is also likely that some patients with so-called “idiopathic hyperprolactinemia” harbor microadenomas that are beyond the resolution of current standard clinical MRI, and these may be identified by Met-PET.

An important limitation of this study is the small sample size. However, the cases reported here represent consecutive patients referred to our tertiary center over a four-year period and, importantly, all Met-PET scans demonstrated unequivocal findings. Although outcomes following TSS and SRS are only available for six patients, all demonstrated clinical and biochemical responses that confirm the accuracy of the PET. A further three patients are awaiting surgery (delayed by the pandemic), and the remaining four patients were all offered surgery. Accordingly, there was no selection bias when referring for surgery, and it seems unlikely that these patients would have fared less favorably at surgery given the comparable Met-PET findings. However, it will be important to reproduce our findings in larger cohorts in a mutlicenter study. In addition, T2 MR sequences were not available in our patients, but may have allowed the identification of some occult tumors as previously reported [67, 68]. Accordingly, future studies should also include a comparison of the performance of T2 MRI with Met-PET. Currently, the restricted availability of ^11^C-methionine (with its short half-life of 20 min) is an important limitation in making this technique more widely available [32, 65], but several other centers in the UK and Europe have recently established molecular imaging for pituitary adenomas, including using related tracers [e.g. ^18^F-fluoroethyltyrosine (^18^F-FET)] and the establishment of a small number of centers in each country that develop appropriate expertise would be consistent with the broader Pituitary Tumor Centre Of Excellence (PTCOE) model [69].

In summary, when MRI appearances are indeterminate in a patient with a microprolactinoma, it is logical for surgeons and patients to be apprehensive about surgery, especially for a condition where pharmacological therapy has traditionally been considered as first line treatment. However, the findings reported here indicate that Met-PET, a non-invasive technique, can facilitate precise localization of microprolactinomas, including when MRI findings are inconclusive, thereby enabling the surgeon to represent the benefits and risks of surgery more accurately.

## Supplementary Information

Below is the link to the electronic supplementary material.

Supplementary file1 (TIFF 984 KB)
