# Supplementary figures and images for: 11C-methionine PET aids localization of microprolactinomas in patients with intolerance or resistance to dopamine agonist therapy
Source: Pituitary. 2022 May 24;25(4):573–86. doi: 10.1007/s11102-022-01229-9 (PMC9345820; doi:10.1007/s11102-022-01229-9)

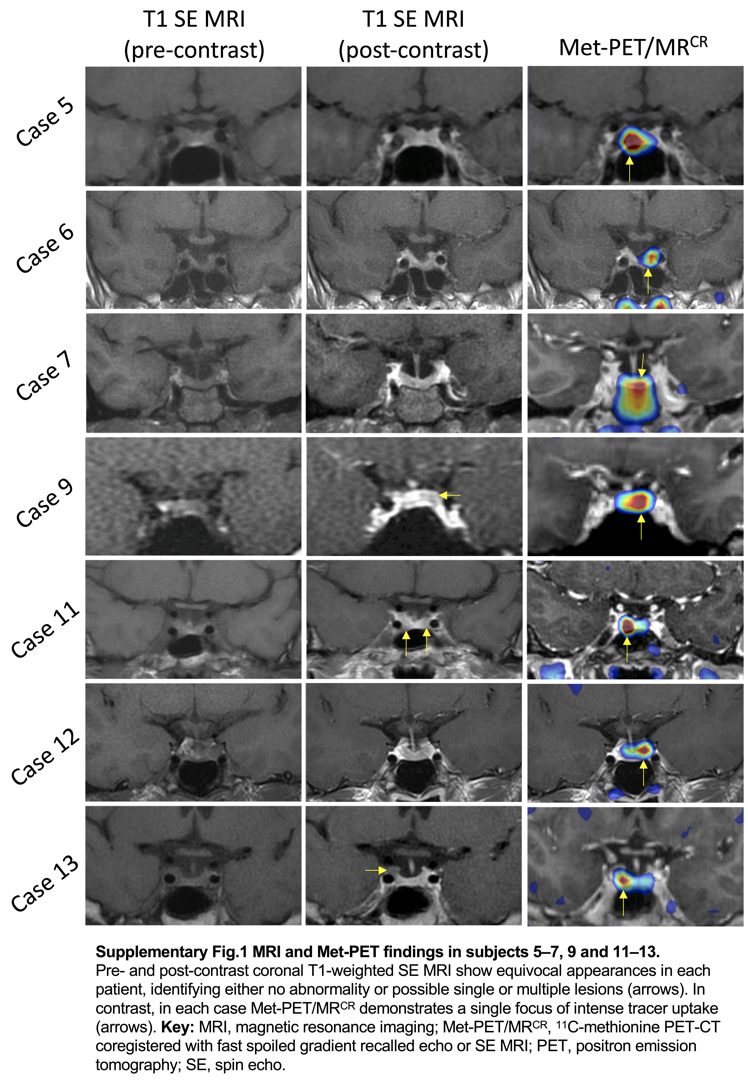

Supplement: Supplementary file 1 — Supplementary file1 (TIFF 984 KB) [file 11102_2022_1229_MOESM1_ESM.tiff]
